# Supplementary material for: Comparing multiple comparisons: practical guidance for choosing the best multiple comparisons test
Source: PeerJ. 2020 Dec 4;8:e10387. doi: 10.7717/peerj.10387 (PMC7720730; doi:10.7717/peerj.10387)
Supplement: Supplemental Information 1 [file peerj-08-10387-s001.docx]

**Supplemental Figures**

**S.I. Figure 1. Type I errors in simulations.** Proportion of Type I experiment-wise error rates (EERs) between the nine multiple comparison tests (MCTs) in each of the four simulation treatments. Simulation group abbreviations can be found in the Figure 1 caption.

**S.I. Figure 2. Type II errors in simulations.** Proportion of Type II experiment-wise error rates (EERs) between the nine multiple comparison tests (MCTs) in each of the four simulation treatments. Simulation group abbreviations can be found in the Figure 1 caption.
